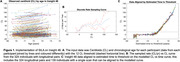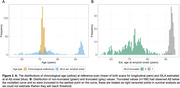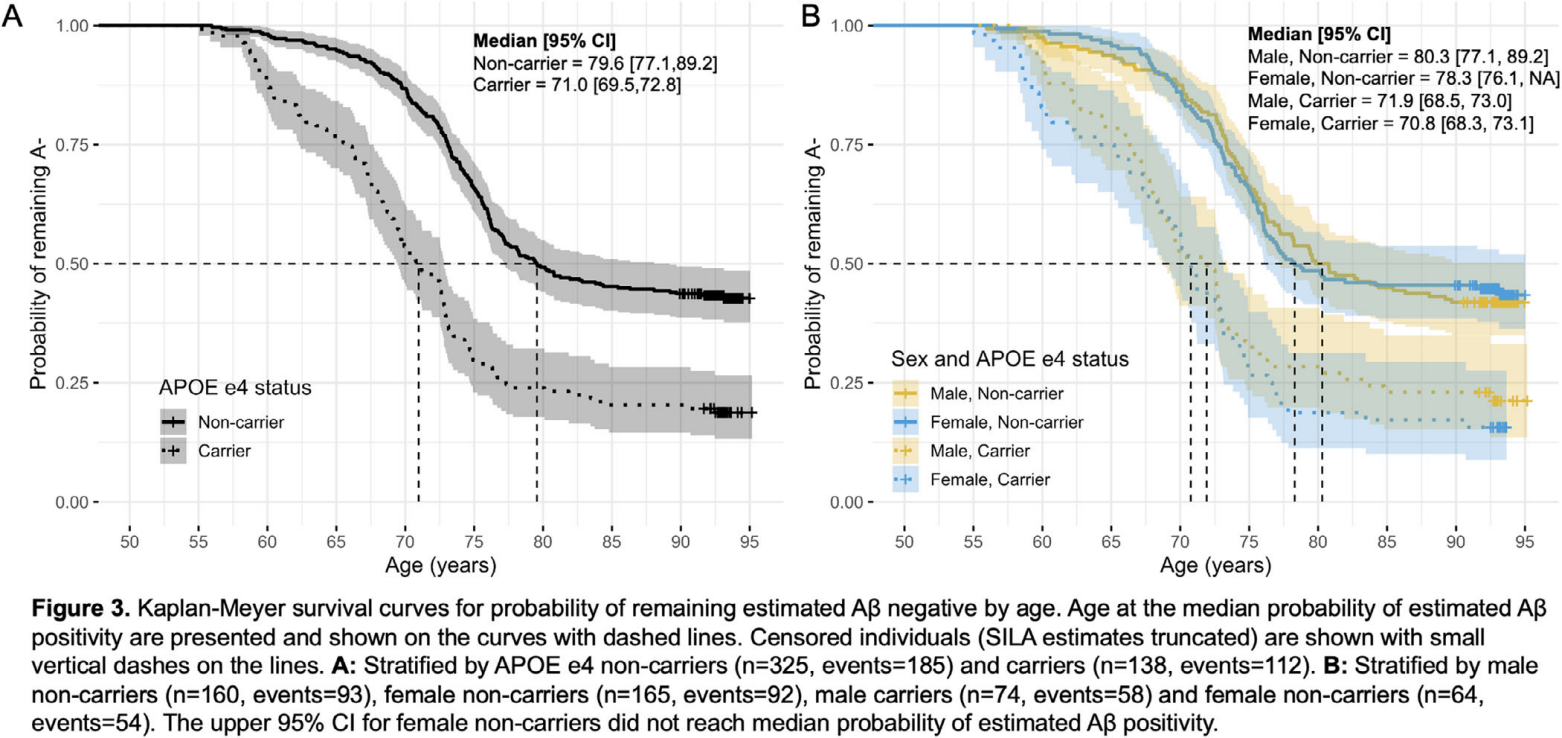# Estimating age at Aβ onset in the 1946 British birth cohort: exploring the influence of APOE ɛ4 carriership and sex

**DOI:** 10.1002/alz.092486

**Published:** 2025-01-09

**Authors:** William Coath, Tobey J. Betthauser, Jordan P Teague, Catherine J Scott, David L Thomas, John Dickson, Pawel J Markiewicz, Frederik Barkhof, Michael Schöll, Marcus Richards, Nick C Fox, David M Cash, Jonathan M Schott, the Insight 46 team

**Affiliations:** ^1^ Dementia Research Centre, UCL Queen Square Institute of Neurology, University College London, London UK; ^2^ University of Wisconsin‐Madison School of Medicine and Public Health, Madison, WI USA; ^3^ Alzheimer’s Disease Research Center, University of Wisconsin‐Madison, Madison, WI USA; ^4^ Department of Medicine, University of Wisconsin‐Madison School of Medicine and Public Health, Madison, WI USA; ^5^ Wisconsin Alzheimer’s Disease Research Center, University of Wisconsin‐Madison, Madison, WI USA; ^6^ Institute of Nuclear Medicine, University College London Hospitals, London UK; ^7^ Dementia Research Centre, UCL Queen Square Institute of Neurology, London UK; ^8^ Dementia Research Centre, Department of Neurodegenerative Disease, UCL Queen Square Institute of Neurology, University College London, London UK; ^9^ Brain Repair and Rehabilitation, UCL Queen Square Institute of Neurology, London UK; ^10^ Wellcome Centre for Human Neuroimaging, UCL Queen Square Institute of Neurology, London UK; ^11^ UCL Institute of Nuclear Medicine, London UK; ^12^ UCL Centre for Medical Image Computing, London UK; ^13^ Institutes of Neurology and Healthcare Engineering, University College London, London UK; ^14^ Amsterdam Neuroscience, Vrije Universiteit Amsterdam, Amsterdam UMC, Amsterdam Netherlands; ^15^ VU University Medical Center, Amsterdam Netherlands; ^16^ Department of Psychiatry and Neurochemistry, Institute of Neuroscience and Physiology, The Sahlgrenska Academy, University of Gothenburg, Mölndal Sweden; ^17^ MRC Unit for Lifelong Health & Ageing at UCL, London UK; ^18^ UK Dementia Research Institute at UCL, London UK

## Abstract

**Background:**

Cerebral Aβ accumulates decades before symptom onset in AD. Sampled iterative local approximation (SILA, Betthauser et al. 2022) is a technique for estimating time from Aβ positivity (Aβ+) using Aβ‐PET. Here we explore the influence of APOE‐ɛ4 carriership and sex on estimated age of Aβ+ (EAAβ) in primarily cognitively normal individuals from the longest‐running British birth cohort.

**Method:**

Participants in Insight 46 were drawn from the 1946 British birth cohort and underwent combined PET/MR scanning with [18F]florbetapir at two timepoints. Centiloid (CL) values were calculated using a whole cerebellum reference region and partial volume correction. We used Gaussian mixture modelling (99^th^ percentile of lower distribution) to define an Aβ+ threshold of 12 CL. We constructed a CL vs Aβ time curve with SILA from available longitudinal Aβ‐PET scans (n=324, interval=2.4+/‐0.2yrs), which was applied to the whole sample (n=463) to calculate EAAβ. Some scans were below the CL range that could be modelled, and the estimated EAAβ was truncated to the furthest time before onset on the CL vs time curve. Kaplan‐Meyer plots and Cox regression were used to investigate EAAβ and risk of estimated Aβ+ by APOE‐ɛ4 and sex, right censoring participants where EAAβ was truncated.

**Result:**

Mean (SD) chronological age was 70.6+/‐0.7yrs at baseline and 73.0+/‐0.6yrs at follow‐up scan and 30.5% were Aβ+ve by follow‐up (n=141, 55.3% APOE‐ɛ4, 51.8% female). SILA results and distribution of EAAβ are shown in Figures 1 and 2. EAAβ were truncated due to low Aβ binding in 35.7% (n=166, 15.7% APOE‐ɛ4, 50% female). APOE ɛ4 carriers reached median EAAβ 8.6 years earlier than non‐carriers (Figure 3A). Median EAAβ for females was marginally earlier than males with overlapping CIs (Figure 3B). APOE‐ɛ4 was strongly associated with risk of estimated Aβ+ (HR[95%CI] = 2.4[1.9,3.0]), adjusted for sex. Female sex (1.1[0.8,1.3]) and the interaction between APOE‐ɛ4 and sex (1.2[0.8,1.9]) were non‐significant.

**Conclusion:**

Estimated Aβ positivity was reached on average 8.6 years earlier for APOE ɛ4 carriers. Consistent with results from other cohorts, APOE‐ɛ4, not sex, significantly heightened the risk of estimated Aβ positivity. Future research will explore life course and downstream events related to Aβ timing.